# Platelets as regulators of anti-tumor immunity: mechanisms and clinical implications

**DOI:** 10.1007/s10555-026-10362-x

**Published:** 2026-08-05

**Authors:** Martin Schlesinger

**Affiliations:** 1 Department of Pharmacy, University of Bonn, Bonn, Germany; 2 Federal Institute for Drugs and Medical Devices (BfArM), Bonn, Germany

**Keywords:** Platelets, Cancer, Metastasis, Immune evasion, Natural killer cells, T cells

## Abstract

Platelets are increasingly recognized as important contributors to tumor progression and metastatic dissemination. Platelets facilitate cancer cell survival, their safe transit through the hostile bloodstream, arrest at distant organ sites, and ultimate outgrowth into metastatic lesions. Although multiple platelet-dependent mechanisms have been implicated in cancer progression—including epithelial-mesenchymal transition and the provision of tumor-supportive growth factors—recent studies increasingly highlight immune modulation as a central mechanism underlying platelet-mediated metastasis. Accordingly, this review summarizes current evidence on the molecular and cellular mechanisms by which platelets regulate cytotoxic T lymphocyte and natural killer (NK) cell-mediated anti-tumor immune responses. Special attention is given to platelet-associated immune checkpoint pathways, including the expression and intercellular transfer of Programmed Death-Ligand 1 (PD-L1), as well as to the release of immunomodulatory mediators such as transforming growth factor-β1 (TGF-β1), thromboxane A₂ (TXA₂), and adenosine generated by platelet ectoenzymes. Finally, the potential implications of platelet-immune interactions for cancer therapy are discussed, with a particular focus on their relevance for immunotherapeutic strategies and metastatic disease control.

## Introduction

Platelets are anucleate cellular fragments derived from bone marrow megakaryocytes that continuously patrol the vasculature and play an essential role in maintaining vascular integrity and hemostatic balance. Despite their small size, platelets express a broad repertoire of adhesion and signaling receptors that enable rapid arrest at sites of vascular injury and the formation of stable hemostatic plugs. Initial platelet recruitment is mediated by the GP Ib-V-IX complex and GPVI, which bind exposed von Willebrand factor and collagen, respectively, thereby initiating platelet activation. Subsequently, integrins such as α_IIb_β_3_ stabilize platelet aggregates through fibrinogen bridging. Platelets can be activated by thrombin, soluble mediators like ADP or thromboxane A₂ (TXA₂), or receptor ligation [1]. Activated platelets release the contents of α- and dense granules, which contain a broad spectrum of bioactive mediators, including ADP, TXA₂, and serotonin, thereby amplifying platelet activation and propagating thrombus formation. Beyond their canonical role in hemostasis, platelets are increasingly recognized as versatile immune modulators that shape both innate and adaptive immune responses through direct cellular interactions and the release of immunoregulatory mediators [2, 3]. In addition, platelets constitutively release extracellular vesicles, which account for the majority of circulating microvesicles in healthy individuals [4, 5]. These vesicles retain platelet-associated surface molecules, including P-selectin and phosphatidylserine, and exert potent procoagulant functions [6]. Moreover, they contribute to neutrophil activation at least in part, through transfer of α_IIb_β_3_ to neutrophils and subsequent activation of NF-κB signaling [7, 8]. Platelet-derived microvesicles can also mediate leukocyte-leukocyte interactions under flow conditions, thereby potentially amplifying inflammatory responses [9, 10]. Moreover, they are capable of transferring a broad range of platelet membrane receptors—including integrin α_IIb_β_3_, GPIbα, P-selectin, and the chemokine receptor CXCR4—to hematopoietic progenitor cells and monocytes, thereby functionally reprogramming recipient cells [10, 11]. In addition, CD40L-containing platelet microvesicles have been shown to promote B cell proliferation and antibody production in murine models [12]. In cancer, platelet-derived microvesicles have been implicated in multiple stages of metastatic progression, including enhancement of tumor cell invasiveness, proliferation, endothelial adhesion, and angiogenesis [13–17]. Nevertheless, their precise contribution to tumor progression remains less well characterized than that of intact platelets. By contrast, direct platelet-tumor cell interactions, largely mediated by platelet adhesion receptors, are now recognized as critical drivers of cancer progression, supporting virtually every stage of the metastatic cascade from primary tumor evolution to colonization of distant organs. For liver cancer, platelets proved critical for the development of non-alcoholic steatohepatitis, liver cirrhosis, and the subsequent development of hepatocellular carcinoma [18]. Cancer cells that leave the primary tumor and enter the blood circulation immediately interact with platelets and activate them through multiple mechanisms. Platelets can induce an epithelial-to-mesenchymal transition in tumor cells [19] and recruit myeloid cells to tumor cell-platelet aggregates, thereby enhancing tumor cell invasiveness [20]. Furthermore, platelets mediate tumor cell arrest at the vascular wall via a wide array of adhesion receptors and promote disruption of the endothelial barrier, facilitating tumor cell extravasation and the formation of metastatic foci in distant organs [21]. Angiogenesis within metastatic lesions is further supported by the release of numerous platelet-derived factors, including pro-angiogenic mediators such as vascular endothelial growth factor (VEGF) and basic fibroblast growth factor (bFGF) [22]. These mechanisms have been comprehensively summarized in previous reviews [23, 24].

As early as 1968, Gasic and colleagues reported that experimental thrombocytopenia profoundly reduces metastatic dissemination in mice, thereby establishing one of the earliest functional links between platelets and metastasis [25]. However, the mechanistic basis of this observation remained unresolved for decades until Nieswandt and colleagues demonstrated that platelets directly shield tumor cells from NK cell-mediated cytotoxicity both in vitro and *in vivo* [26]. Upon activation by tumor cells, platelets rapidly adhere to and cloak circulating tumor cells, thereby establishing a physical barrier that limits NK cell recognition and cytolytic engagement. This seminal mechanism, observed across multiple mouse strains and tumor models, is now considered a fundamental pathway by which platelets protect circulating tumor cells from immune surveillance. Importantly, subsequent studies have revealed that platelet-mediated immune evasion extends far beyond physical shielding and involves a broad spectrum of molecular and immunoregulatory mechanisms that actively suppress antitumor immunity. Notably, in recent years, research has increasingly focused on the immunological functions of platelets. On a molecular level, Kopp and colleagues demonstrated that platelet-derived transforming growth factor-β1 (TGF-β1) downregulates NKG2D expression on natural killer (NK) cells, resulting in impaired NK cell-mediated anti-tumor activity [27]. Similarly, the transfer of major histocompatibility complex (MHC) class I molecules from platelets to tumor cells has been identified as a fundamental immune evasion mechanism that reduces NK cell cytotoxicity [28]. More recently, substantial progress has been made in the identification and functional characterization of platelet surface receptors that contribute to the suppression of tumor-specific immune responses. In addition, platelet-derived soluble mediators have been shown to locally modulate immune cell function within the tumor microenvironment, and platelet-associated ectoenzymes catalyze the generation of immunosuppressive adenosine. This review summarizes recent advances in the understanding of platelet-mediated suppression of immune surveillance against cancer cells, addressing a timely and clinically relevant aspect of cancer biology that has not yet been comprehensively reviewed.

### PD-L1

Programmed death ligand 1 (PD-L1, CD274) is a central immune checkpoint molecule expressed across a wide spectrum of human malignancies [29, 30]. Engagement of surface-expressed PD-L1 with programmed cell death protein 1 (PD-1) on T lymphocytes suppresses cytotoxic T-cell activity, thereby facilitating immune evasion by tumor cells [31]. Therapeutic blockade of the PD-1/PD-L1 axis using immune checkpoint inhibitors has fundamentally transformed the treatment landscape of several cancer entities [31, 32]. Notably, a subset of patients achieves durable clinical responses, including long-term remission with sustained disappearance of metastatic lesions even after treatment discontinuation [33]. Nevertheless, a considerable proportion of patients fails to derive benefit from immune checkpoint blockade, and the molecular determinants underlying therapeutic response or resistance remain incompletely understood.

Accordingly, the identification of predictive biomarkers and mechanistic pathways that attenuate PD-1/PD-L1 blockade is of critical importance to overcome therapeutic resistance and to enable rational patient stratification. More recently, PD-L1 expression has been identified on platelets, adding an additional layer of complexity to the cellular targets of PD-L1-directed therapies and introducing platelets as potential contributors to immune evasion, relapse, or therapeutic resistance [34]. Elevated platelet-associated PD-L1 levels have been reported in patients with head and neck squamous cell carcinoma as well as in smokers [34]. Moreover, PD-L1 has been detected on platelet-derived extracellular vesicles released upon platelet activation by agonists such as ADP or thrombin [35]. Platelet PD-L1 was shown to play a minor role in platelet aggregation and thrombus formation [36].

Given that platelets express PD-L1, they may substantially influence antitumor immunityby suppressing T cell effector functions and potentially modulating responses to PD-1/PD-L1-directed therapies. Moreover, platelets can also modulate PD-L1 expression on tumor cells.

Battinelli and colleagues demonstrated that platelets can induce PD-L1 expression on breast and lung cancer cells through the release of high-molecular-weight epidermal growth factor (EGF), which engages epidermal growth factor receptors on tumor cells. Platelets express pro-EGF in the membranes that is cleaved by ADAM10 (A Disintegrin and metalloproteinase domain-containing protein 10) upon platelet activation and released as high-molecular-weight EGF. High-molecular-weight EGF is biologically active and fosters PD-L1 expression whereas other cytokines that are highly abundant in platelet granules (e. g. CXCL5, CCL5, IL-8 and IL-6) are surprisingly not responsible for the PD-L1 upregulation. The increased expression of PD-L1 dampened the cytotoxic T-cell function against breast cancer cells and this effect was rescued by blockade of the EGF receptor with the antibody cetuximab [37]. Thus, the combination of EGFR tyrosine kinase inhibitors (e. g. gefitinib, erlotinib, afatinib, or osimetrinib) with PD-L1 blocking antibodies could have synergistic effects and resulting in a favorable patient outcome. However, this should first be thoroughly tested in clinical trials.

A complementary mechanism of platelet-mediated PD-L1 regulation was described by Schneider and colleagues, implicating serotonin released from platelet dense granules.

They found an enhanced infiltration of CD8^+^ T cells in subcutaneous and orthotopically injected murine pancreatic and colorectal cancers in serotonin-deficient mice which was accompanied by a reduced tumor growth and reduced PD-L1 expression [38]. Serotonin elevated the expression of PD-L1 on mouse and human cancer cells in vitro. Concentrations of serotonin in metastases of patients with abdominal tumors inversely correlated with CD8^+^ T cell infiltration. The combination of a pharmacological serotonin depletion with a PD-1 checkpoint inhibitor induced a long-term tumor control in mice with colorectal and pancreatic tumors. Thus, these data add another mechanism by which platelets modulate the PD-L1 expression of cancer cells and thereby promote tumor progression. Further, the combination of a checkpoint and platelet inhibition to mitigate serotonin release seems to hold promise to enhance the anti-tumor immunity.

An additional platelet-tumor interaction affecting PD-L1 dynamics was identified by Hinterleitner and colleagues, who demonstrated that non–small cell lung cancer cells can transfer PD-L1 to platelets through direct contact involving platelet GPIbα, integrin α_5_β_1_, and fibronectin [39]. Surprisingly, the transient contact does not activate the platelets, but is obviously sufficient for the PD-L1 translocation process. This is an interesting finding, since the direct contact with cancer cells is traditionally considered to have a strong activatory impact on platelets, a process that is known as tumor cell induced platelet activation (TCIPA). Notably, PD-L1 presenting platelets from NSCLC patients reduced the activity of effector memory T cells in vitro and in patients with high levels of platelet PD-L1, a reduced number of T cells in the tumor microenvironment was detectable. Furthermore, the authors developed a method to calculate expression of PD-L1 in unstimulated and pre-activated platelets to avoid in vitro manipulation. The calculated platelet PD-L1 levels correlated with NSCLC patients´ overall survival, the occurrence of metastases in liver and bone and the response to a platinum-based chemotherapy as well as disease activity and response to immune-checkpoint inhibitor therapy [39]. The platelet PD-L1 expression status turned out to have a higher predictive value compared to conventional histological PD-L1 detection. In this context, a blockade of PD-L1 with the antibody atezolizumab was associated with a longer progression-free survival especially in those NSCLC patients with high expression of platelet PD-L1 [40].

**Fig. 1:**
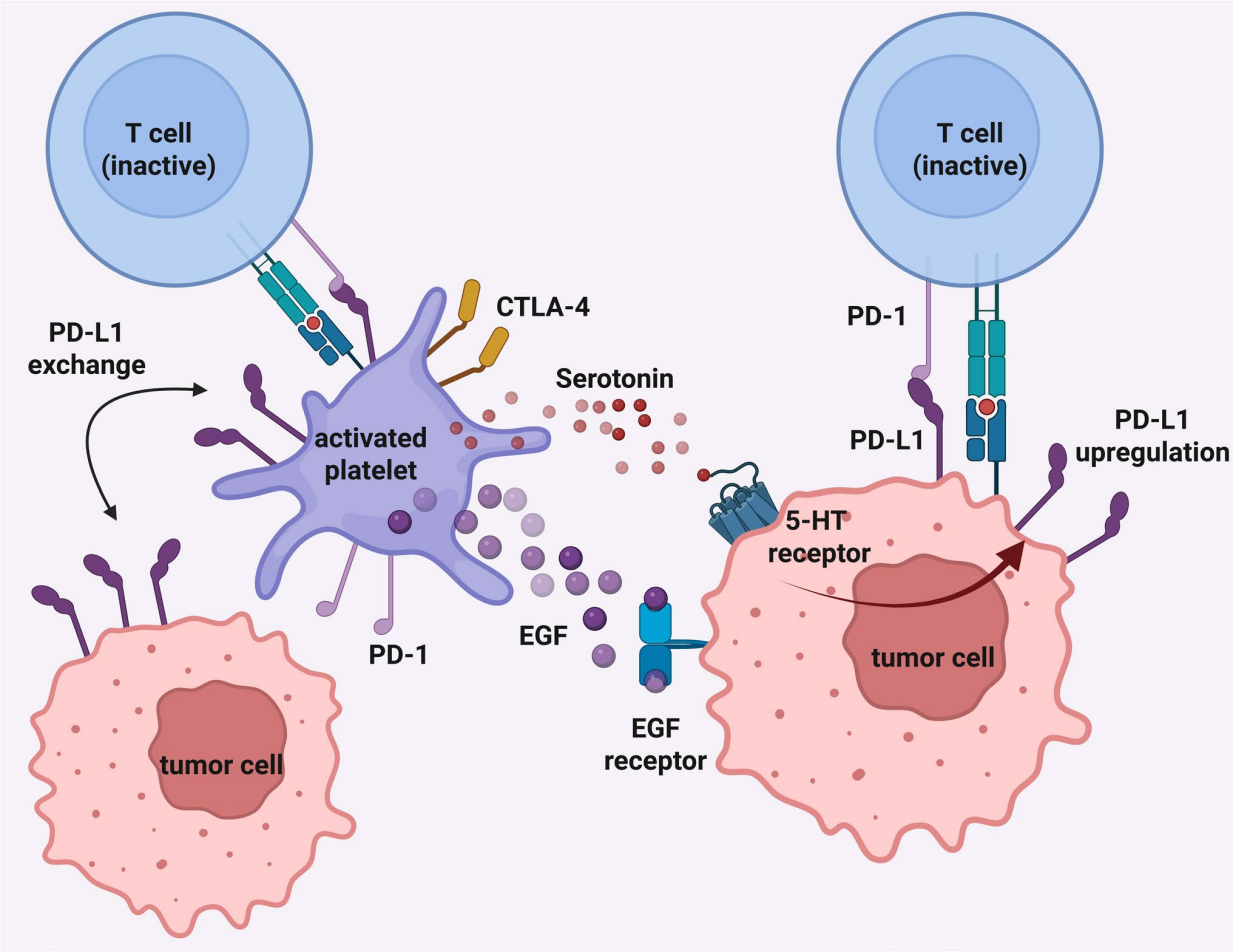
Activated platelets contribute to the regulation of anti-tumor T cell responses through multiple PD-L1-dependent mechanisms. Platelets can acquire PD-L1 from tumor cells or, conversely, transfer PD-L1 to tumor cells, thereby enhancing immune evasion. In addition to direct PD-L1 exchange, activated platelets release soluble mediators, including epidermal growth factor (EGF) and serotonin, which induce PD-L1 expression on tumor cells. Collectively, these processes contribute to the suppression of cytotoxic T cell function and promote an immunosuppressive tumor microenvironment

In contrast, Zaslavsky et al. reported that platelets intrinsically express PD-L1 within intracellular granules, which is rapidly translocated to the platelet surface upon activation. The interaction of PD-L1 negative tumor cells with platelets led to a PD-L1 transfer from the platelets to the cancer cells like previously shown for MHC-I receptors or integrin β_3_ subunits [41, 42]. Co-incubation of platelets with different cancer cell entities resulted in reduced T cell induced cytotoxicity towards the tumor cells and this effect was reversed by addition of a PD-L1 neutralizing antibody or platelet inhibition with aspirin. The growth of PD-L1 negative tumors in PD-L1^−/−^ mice was dramatically enhanced following repeated transfusion of PD-L1 positive platelets [41]. Thus, platelet PD-L1 seems to have a strong impact on the immune surveillance in the tumor microenvironment and tumor progression. The different functions of platelet PD-L1 are illustrated in Fig. 1.

These findings also explain why an immune-checkpoint inhibitor therapy benefits patients with PD-L1 negative tumors. This fact was observed in clinical practice and trials but the underlying mechanism remained ambiguous [43–45]. This is corroborated by data from Schaubaecher and colleagues in a model of triple negative breast cancer [46]. They revealed that apoptotic/necrotic tumor cells release oligonucleotides that recruit platelets into the tumor tissue which subsequently are activated by GPVI-mediated binding to collagen in the tumor microvasculature. These procoagulant platelets present PD-1 and CTLA-4 (CD152) on their membrane and induce a protumorigenic microenvironment. Blockade of PD-1 and CTLA-4 enhanced tumor infiltration with antitumorigenic cytotoxic T lymphocytes and TH_1_ cells and simultaneously decreased infiltration of protumorigenic neutrophils and classical monocytes finally resulting in a reduced tumor growth. To further explore, how platelets contact and modulate the immune cell function on a molecular level, the authors identified platelet GPIbα as the relevant receptor that binds to von Willebrand Factor decorated CD3 T cells and mediates the close interaction. Consequently, a blockade of GPIbα showed similar tumor-suppressive effects in a breast cancer mouse model as a simultaneous PD-1 and CTLA-4 inhibition. Collectively, these studies demonstrate that platelet-expressed immune checkpoint molecules critically shape immune cell infiltration within the tumor microenvironment, thereby exerting either pro- or antitumorigenic effects. Furthermore, this novel data also may help explain why patients who do not express PD-L1 or other immune checkpoint molecules on their tumors benefit from an immune checkpoint inhibitor therapy. However, some patients do not profit from immune checkpoint inhibitors at all. It can be speculated that tumors of those patients do not trigger a platelet PD-L1 expression or that other immunoregulatory pathways are at play that bypass the blockade of platelet expressed immune checkpoint molecules. Furthermore, these findings raise the possibility that combining PD-L1 blockade with concomitant platelet inhibition could provide therapeutic benefit in selected cancer settings, although this concept remains to be validated in clinical studies.

### Erbin-mediated mitochondrial crosstalk between platelets and B cells

Erbin (ERBB2 interacting protein) was identified as an adaptor for the ERBB2/HER2 receptor in epithelial cells and contains 16 leucine-rich repeats in its amino terminus and a PDZ domain at its C-terminus [47, 48]. Erbin localizes to the lateral membrane of polarized epithelial cells and is broadly expressed across multiple human tissues [47, 49]. Functionally, Erbin regulates multiple signaling pathways, including RAS/RAF/ERK, TGF-β/Smad, and Nod2 signaling [50]. In cancer biology, Erbin has been reported to exert context-dependent effects, functioning either as a tumor suppressor or as a modulator of tumor progression depending on cellular context [50]. Recent work by Zhang and colleagues uncovered a previously unrecognized role for platelet-derived Erbin in colorectal cancer (CRC) progression and metastasis [51]. CRC patients with metastasis exhibited a significantly increased platelet count and their platelets had elevated Erbin expression levels compared with patients without metastatic lesions or healthy individuals. Notably, metastatic CRC patients displayed both increased platelet counts and elevated platelet Erbin expression compared with non-metastatic patients and healthy controls. To investigate its functional relevance, global and platelet/megakaryocyte-specific Erbin knockout mouse models were generated. Platelets lacking Erbin exhibited increased aggregatory capacity and markedly suppressed lung metastasis. At the immune level, Erbin deficiency reduced pulmonary PD-1⁺ and PD-L1⁺ CD4⁺ and CD8⁺ T cells while increasing the frequency of IFNγ⁺ and perforin⁺ effector T cells. In parallel, pulmonary CD138⁺ plasma cells were markedly increased in Erbin-deficient mice. Comparable immune alterations were observed following adoptive transfer of Erbin-deficient platelets into tumor-bearing mice. Adoptive transfer of B cells from Erbin-deficient mice similarly reduced CRC metastasis and diminished T cell exhaustion. In vitro, Erbin-deficient platelets alone exerted only minor effects on T cell cytotoxicity.

**Fig. 2:**
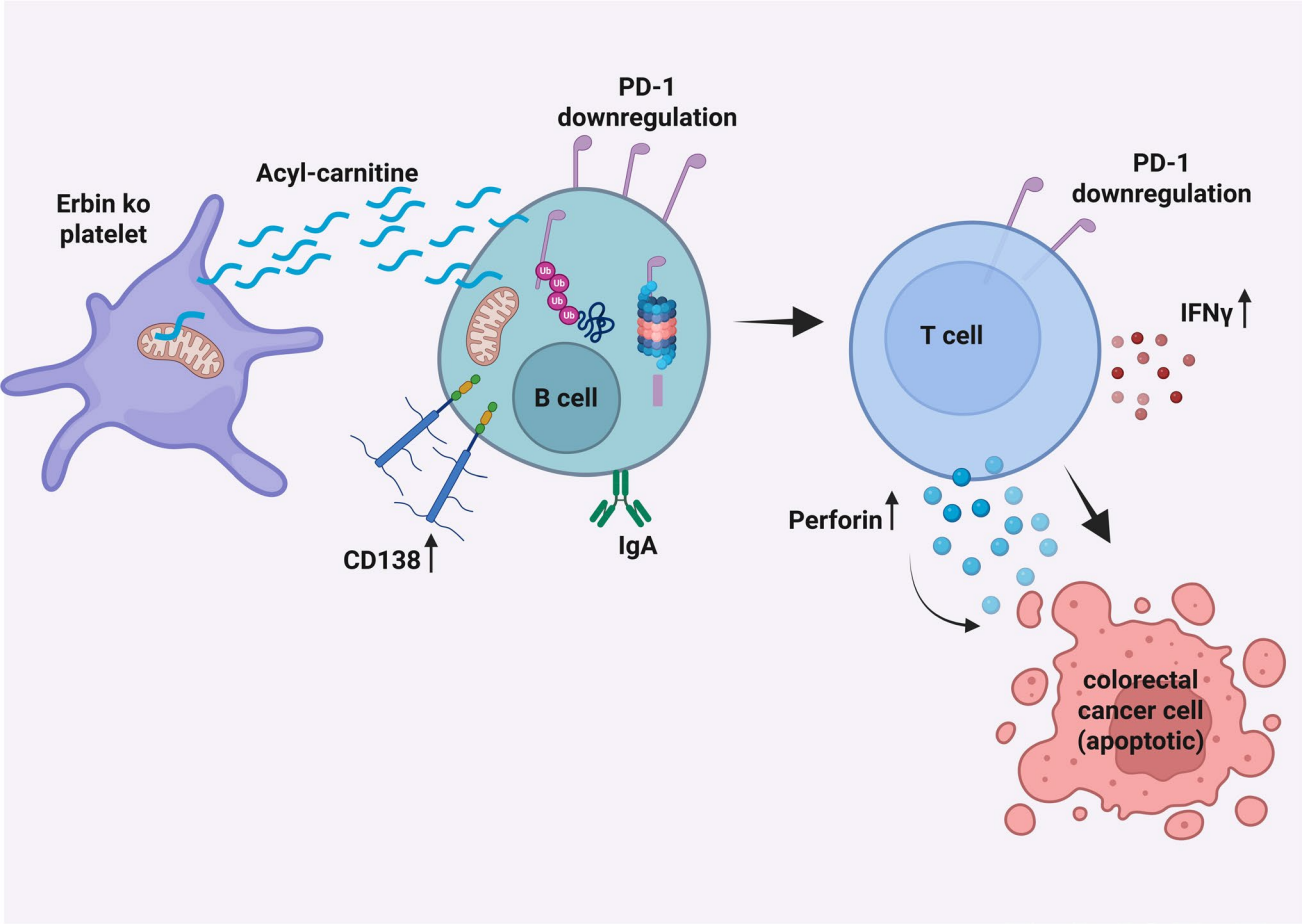
Platelets lacking Erbin secrete increased levels of palmitoyl-L-carnitine, which has been shown to promote ubiquitination and subsequent degradation of PD-L1 on B cells. This reduction in PD-L1 expression is associated with enhanced T cell effector function, including increased secretion of interferon-γ (IFN-γ) and perforin, resulting in augmented anti-tumor activity

However, inclusion of B cells markedly enhanced T cell cytotoxicity, indicating that Erbin-deficient platelets potentiate antitumor immunity indirectly via B cell activation. Mechanistically, Erbin deficiency increased mitochondrial size and activity in platelets. Consistently, mitochondrial respiratory chain activity and expression of enzymes involved in acyl-carnitine β-oxidation and transport were elevated in Erbin-deficient platelets and megakaryocytes. Targeted lipidomic analyses revealed increased acyl-carnitine levels in Erbin-deficient platelets, platelet supernatants, and lung metastases. Exposure of B cells to palmitoyl-L-carnitine (PLC) promoted differentiation into CD138⁺ plasma cells while reducing PD-1-expressing plasma cell subsets. PLC further enhanced fatty acid β-oxidation, oxygen consumption, and mitochondrial remodeling in splenic B cells. Mechanistically, PLC promoted binding of the E3 ubiquitin ligase FBXO38 to PD-1, facilitating PD-1 ubiquitination and degradation. *In vivo* targeting of Erbin in platelets and megakaryocytes using a nanovesicle delivery system markedly suppressed CRC lung metastasis and increased pulmonary CD138⁺ plasma cells and IFNγ⁺/perforin⁺ CD4⁺ and CD8⁺ T cells while reducing PD-1-expressing immune cells [51]. Collectively, these findings identify platelet- and megakaryocyte-derived Erbin as a regulator of metastatic progression through modulation of antitumor immunity. Importantly, Erbin-deficient platelets enhance immune responses indirectly by metabolically reprogramming B cells, which subsequently amplify cytotoxic T cell activity (Fig. 2). This platelet-B cell-T cell signaling axis highlights an additional layer of complexity in platelet-mediated immune regulation within the metastatic niche.

### Impact of platelet-derived TXA_2_ on anti-tumor T cell function

Early experimental observations demonstrating that nonsteroidal anti-inflammatory drugs attenuate the development of chemically induced tumors in murine models led to the hypothesis that cyclooxygenases (COX) contribute to carcinogenesis [52]. Given that aspirin irreversibly inhibits COX-1 at low doses and COX-2 at higher concentrations, extensive epidemiological studies were initiated to assess whether this long-established drug might confer therapeutic or preventive benefits in oncology.

Meta-analyses of large observational cohorts comprising approximately 250,000 patients across 18 cancer entities revealed an association between aspirin use and an approximately 20% reduction in cancer-related mortality [53, 54]. These observations were supported by randomized clinical trials in patients with PIK3CA-mutant colorectal cancer, in whom low-dose aspirin significantly reduced disease recurrence [55, 56].

By contrast, a more recent randomized, double-blind, placebo-controlled trial failed to demonstrate a significant improvement in disease-free survival with aspirin treatment [57]. Moreover, in the context of cancer prevention in otherwise healthy individuals, long-term aspirin use remains controversial, as reported benefits were modest or absent [58, 59] and, in some studies, outweighed by adverse outcomes [60, 61]. However, several studies are ongoing or currently recruiting to further elucidate the impact of a COX blockade on cancer outcome. On the molecular level, it was exhibited that aspirin can reverse a platelet-mediated cancer cell proliferation and upregulation of the oncoprotein c-MYC [62].

TXA₂ is a bioactive eicosanoid derived from arachidonic acid through the sequential enzymatic activity of phospholipase A₂, COX-1/COX-2, and thromboxane synthase. Initially identified as a platelet-derived mediator, TXA₂ is now recognized to be produced by multiple cell types, including macrophages, neutrophils, and endothelial cells, thereby extending its functional relevance beyond hemostasis [63].

In the context of anti-tumor immunity, Yang and colleagues provided a mechanistic explanation for the anti-metastatic effects of aspirin and selective COX-1 inhibition [64].

They demonstrated that platelet-derived TXA₂ engages the thromboxane prostanoid (TP) receptor on T cells, resulting in activation of the RHO guanine nucleotide exchange factor ARHGEF1. This signaling cascade increases RHOA-GTP levels and RHO kinase activity, promotes PTEN activation, and ultimately suppresses AKT and S6 phosphorylation. Importantly, AKT and S6 phosphorylation are essential for robust T-cell activation, cytokine production, and effective anti-tumor immune responses [64].

Accordingly, pharmacological inhibition of platelet COX-1 by aspirin or genetic ablation of COX-1 in platelets and megakaryocytes markedly reduced TXA₂ secretion and significantly decreased metastatic burden in the lungs and liver in murine models.

In contrast, an inhibition of COX-2 did not affect metastasis. Interestingly, a platelet blockade with an inhibitor of the purinergic receptor P2Y12, which is involved in ADP-mediated platelet activation, had no effect on pulmonary metastasis. Collectively, these findings clearly highlight platelet-derived TXA_2_ as a potent inhibitor of T cell reactivity against metastasizing cancer cells and open a new avenue for application of aspirin or other COX-1 inhibitors not only for cancer prevention but also after tumor occurrence to interfere in the formation of metastatic foci.

These findings are consistent with earlier work by Lucotti et al., who demonstrated that the platelet COX-1/TXA₂ axis is critical for the formation of a pre-metastatic niche. Furthermore, platelet-derived TXA_2_ fosters the platelet-tumor cell clot formation and the recruitment of pro-metastatic myeloid cells to the intravascular tumor cell aggregates [65]. Aspirin efficiently reduced clustering of monocytes around the intravascular tumor cells and decreased endothelial activation adjacent to platelet tumor cell clots finally reducing the number of metastatic lung colonies.

Similar to the above-described effects of platelet-derived PD-L1 on antitumor immunity, these findings raise the possibility that aspirin-mediated inhibition of platelet-derived TXA₂ may synergize with targeted therapies or immune checkpoint blockade to enhance antitumor immune responses and potentially limit metastatic progression [66]. However, the therapeutic relevance of such combinatorial approaches remains speculative and requires further investigation to define optimal treatment modalities and clinical settings. Importantly, the potential risks associated with COX-1/TXA₂ inhibition, particularly bleeding complications, will need to be carefully evaluated to establish a favorable therapeutic window.

### CD40L

CD40 ligand (CD40L, CD154) is a type II transmembrane protein that belongs to the tumor necrosis factor (TNF) superfamily. CD40L was initially identified on activated CD4^+^ T cells, where it plays an important role in contact-dependent B cell activation, proliferation, and antibody class switching [67, 68]. Beyond T cells, CD40L expression has been identified on multiple hematopoietic and non-hematopoietic cell types, including platelets, which store CD40L in α-granules and rapidly translocate it to the cell surface upon activation [69]. CD40L ligates to its receptor CD40 and to different integrins and triggers different signaling cascades [70]. Beyond lymphocytes, both CD40 and CD40L are expressed by a broad range of hematopoietic and non-hematopoietic cell types, including monocytes, basophils, eosinophils, dendritic cells, fibroblasts, smooth muscle cells, and endothelial cells. Consequently, CD40 ligation orchestrates diverse immune and inflammatory responses, including the induction of adhesion molecules, cytokines, matrix-remodeling enzymes, prothrombotic mediators, and apoptotic signaling pathways [69]. In the hemostatic system, platelet-derived CD40L acts in a paracrine manner via platelet-expressed CD40 and integrins, thereby amplifying platelet activation and contributing to thrombus stabilization [71, 72]. Within the tumor microenvironment, platelet-derived CD40L has been shown to promote the proliferation of intravascular large B cell lymphoma, in line with the central role of CD40-CD40L signaling in B cell activation [73]. Accordingly, a comparable pro-tumorigenic function of platelet CD40L has been proposed for other CD40-positive malignancies [74]. However, accumulating evidence indicates that the role of platelet CD40L in cancer is highly context dependent. In a murine model of primary and metastatic liver cancer, Ma and colleagues reported an opposing, antitumorigenic function of platelet CD40L [75]. In orthotopically transplanted liver tumors, they found that platelet CD40L binds to CD40 on CD8⁺ T cells. This interaction promoted CD8⁺ T cell-mediated antitumor immunity, resulting in reduced tumor growth, whereas pharmacological platelet inhibition increased tumor burden. Taken together, these findings underscore the dual and context-specific role of platelet CD40L in cancer, functioning either as a pro-tumorigenic signal or as a mediator of antitumor immune responses. Thus, platelet-mediated immune modulation appears to be critically shaped by tumor entity, microenvironmental cues, and disease stage. A detailed mechanistic understanding of these context-dependent effects will be essential to identify clinical settings in which platelet-targeted therapies can be safely applied without impairing immune-mediated tumor surveillance.

### TGF-β

The family members of transforming growth factor-beta (TGF-β) exert multiple critical functions in the embryonic development, homeostasis, proliferation, wound healing and differentiation in diverse cell types in health and disease [76]. It is expressed by almost all tissues including salivary glands, muscles, kidneys, liver, heart, and brain [77–79]. Notably, platelets represent one of the most abundant physiological reservoirs of TGF-β among normal tissues [80]. Among the family members, TGF-β1 is predominantly immunosuppressive and represents a central regulator of tumor-associated immune evasion by attenuating both innate and adaptive immune responses [81]. TGF-β1 is synthesized as a latent complex comprising the mature cytokine non-covalently associated with its latency-associated peptide (LAP), thereby requiring extracellular activation to exert biological activity [82, 83]. Activation of latent TGF-β1 is tightly regulated and mediated by specific receptors and proteases, including integrins and the docking receptor glycoprotein A repetitions predominant (GARP) [82, 84]. Platelets have long been recognized as major modulators of innate and adaptive immunity, with TGF-β1 representing one of their most potent immunoregulatory mediators [85, 86].

Notably, TGF-β1 reduces CD8⁺ and NK cell cytotoxicity and converts conventional CD4^+^ to immunosuppressive Treg cells [87, 88]. In line with these immunosuppressive functions, platelet-derived TGF-β1 has emerged as a critical regulator of antitumor immune surveillance. Rachidi and colleagues provided seminal evidence that platelets suppress T cell-mediated antitumor immunity in vitro and *in vivo*, primarily through the release of TGF-β and, to a lesser extent, lactate [89]. Importantly, platelets were identified as the principal source of circulating latent TGF-β (LTGF-β). Platelet-expressed GARP binds LTGF-β and facilitates its conversion into biologically active TGF-β, linking platelet activation directly to systemic immunosuppression; accordingly, dual platelet inhibition prolonged tumor control and relapse-free survival. Mechanistically, thrombin was shown to cleave the GARP-LTGF-β complex from the platelet surface, thereby releasing active TGF-β into the circulation. Blockade of thrombin restrained the systemic release of active TGF-β and led to an intensification of the immune response by increasing tumor-infiltrating CD8⁺ T cells, NK cells and neutrophils in the tumor microenvironment. Consequently, thrombin inhibition enhanced immune cell infiltration and synergized with PD-1 immune checkpoint blockade in preclinical cancer models [90]. Clinically, elevated levels of soluble GARP correlate with disease stage, suggesting that prevention of GARP cleavage may represent a strategy to restore antitumor immunity and overcome immunotherapy resistance. Beyond direct effects on T and NK cells, platelet-derived TGF-β also contributes to the expansion of immunosuppressive myeloid-derived suppressor cells (MDSCs) [91]. A thrombin inhibition reduced platelet activation, TGF-β concentration and the number of splenic MDSCs in mice with 4T1 mammary carcinoma. Given the central role of MDSCs in suppressing T cell responses and promoting tumor progression, platelet-derived TGF-β emerges as a key upstream regulator of systemic immune suppression [92, 93]. Accordingly, platelet-derived TGF-β critically limits the efficacy of T cell-based immunotherapeutic strategies.

Bispecific antibodies that recruit immune cells to tumor cells are commonly used in clinical practice and a multitude of clinical trials are currently ongoing [94, 95]. Nevertheless, durable responses remain rare, largely due to immune exhaustion and adaptive resistance mechanisms [96, 97]. Recent evidence implicates platelets in the development of resistance to T cell-engaging antibodies. Notably, bispecific antibodies bind to and activate T cells which upregulate CD40L expression on the cell surface. CD40L, in turn, activates platelets and leads to secretion of TGF-β from platelet granules. Platelet-derived TGF-β efficiently suppresses T cell reactivity, perforin secretion and finally tumor cell lysis [98]. This platelet-T cell feedback loop establishes a powerful negative regulatory circuit that limits sustained antitumor cytotoxicity. Consequently, combining T cell-engaging antibodies with TGF-β blockade or platelet inhibition may substantially enhance therapeutic efficacy. This inhibitory axis is likely relevant for additional T cell-based therapies, including CAR T cells, warranting further mechanistic investigation [98]. Taken together, accumulating evidence suggests that both intratumoral and systemic TGF-β may constitute important barriers to effective cancer immunotherapy. Given their capacity to attenuate T cell-mediated immunity, platelets may act as relevant modulators of immunotherapeutic efficacy. Collectively, these findings raise the possibility that targeted modulation of platelet reactivity may enhance antitumor immune responses across diverse therapeutic settings. However, the translational potential of this approach remains to be established in appropriate preclinical models and clinical studies.

### Impact of platelets on adenosine metabolism – CD39

**Fig. 3:**
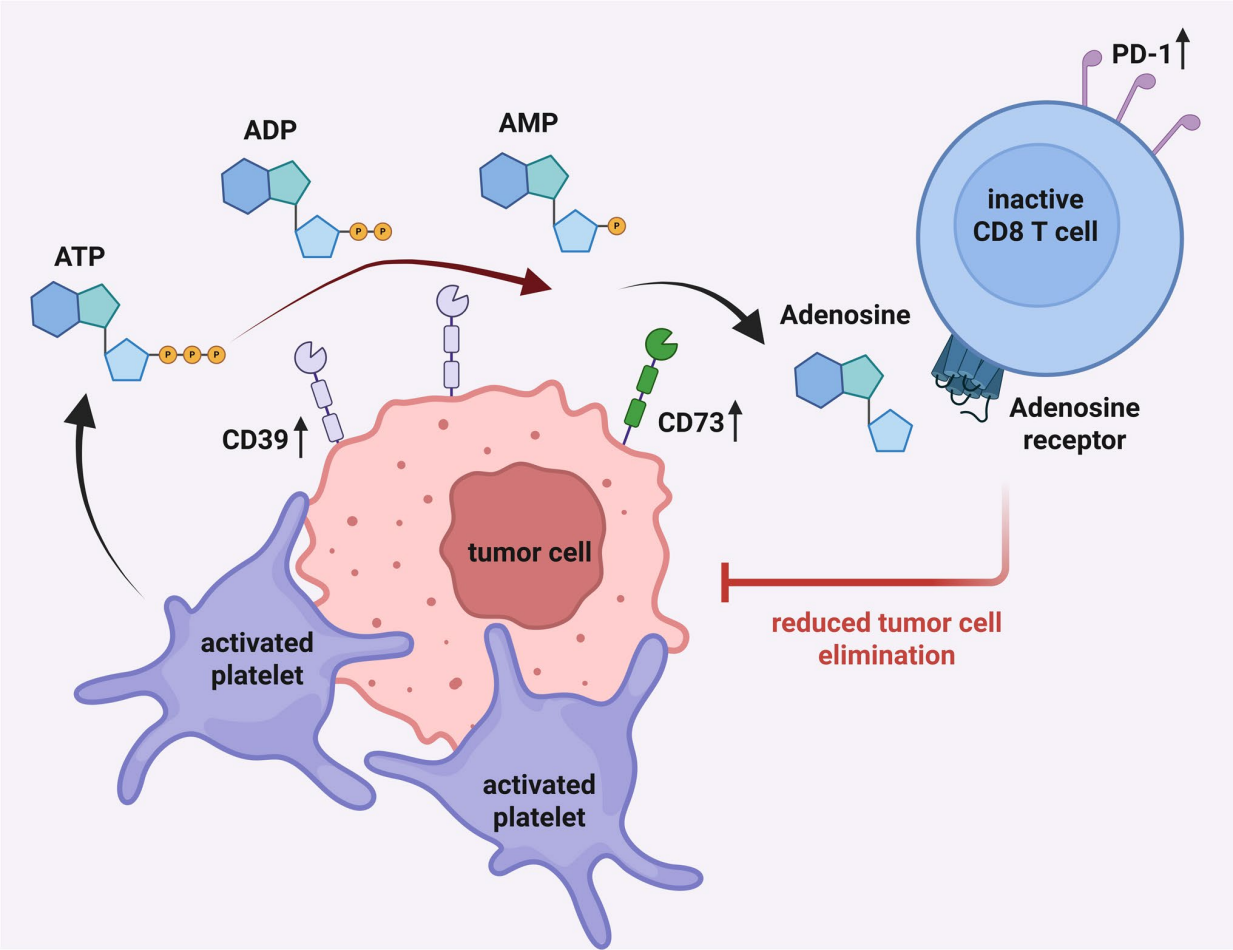
Tumor cell-activated platelets promote the upregulation of the ectonucleotidases CD39 and CD73 on tumor cells. These enzymes catalyze the sequential conversion of extracellular ATP into adenosine, a potent immunosuppressive mediator. Increased adenosine levels in the tumor microenvironment contribute to the suppression of CD8⁺ T cell-mediated anti-tumor responses

In contrast to TGF-β, the release of ATP by dying tumor cells induces the attraction of dendritic cells and T lymphocytes into the tumor bed and elicits a potent inflammatory antitumor immune response [99, 100]. Conversely, enzymatic degradation of extracellular ATP into adenosine establishes a profoundly immunosuppressive tumor microenvironment that promotes tumor immune escape. Notably, adenosine exerts broad immunomodulatory effects on multiple cellular components of the tumor microenvironment, including neutrophils, dendritic cells, NK cells, T cells, macrophages, and MDSCs, respectively. Accordingly, multiple clinical trials are currently evaluating strategies to inhibit adenosine production, block adenosine receptor signaling, or combine adenosine pathway inhibitors with established immunotherapeutic approaches [101]. The adenosine metabolic switch is primarily mediated by the sequential activity of the ectonucleotidases CD39 and CD73. The rate-limiting enzyme CD39 cleaves ATP and ADP to AMP and is expressed on the membranes of different leukocytes, tumor cells, epithelial, vascular endothelial cells as a result of tissue hypoxia, oxidative stress or inflammation [102, 103]. CD73 subsequently converts AMP into adenosine and is expressed by a broad range of stromal, immune, and tumor cells [104]. Adenosine ligates to A1, A2a, A2b, and A3 adenosine receptors and exerts strong immunosuppressive effects in the tumor microenvironment [105]. Through engagement of these receptors, adenosine potently suppresses antitumor immune effector functions (Fig. 3). Accordingly, CD39 and CD73 have emerged as promising immune checkpoint targets in cancer therapy. Recent evidence implicates platelets as critical modulators of this purinergic immune checkpoint axis. In this context, Ning demonstrated that platelets induce CD39 expression on colon and prostate cancer cells [106]. Direct platelet-tumor cell contact, rather than soluble platelet-derived factors, enhanced CD39 expression and promoted tumor cell invasiveness via upregulation of matrix metalloproteinases MMP-2 and MMP-3 [106]. Notably, CD39 induction did not affect tumor cell proliferation or migratory capacity. Functionally, elevated CD39 expression suppressed cytotoxic T cell activity and promoted the expression of inhibitory receptors such as PD-1 on CD8⁺ T cells, thereby fostering an immunosuppressive tumor microenvironment in mice. Earlier studies further demonstrated that platelets can induce CD73 expression across a broad spectrum of tumor cells through direct cellular interactions [107, 108]. Taken together, these findings position platelets as key orchestrators of the ATP-adenosine immune checkpoint by inducing both CD39 and CD73 on tumor cells, thereby shielding malignant cells from immune surveillance. Whether these preclinical findings can ultimately be translated into effective therapeutic strategies in humans remains to be determined and warrants further investigation.

### Influence of platelet mediators on anti-tumor activity of NK cells

Natural killer (NK) cells are central mediators of antitumor immunity, recognizing and eliminating malignant cells through the “missing-self” and “induced-self” paradigms. That means that cells with low expression of MHC class I epitopes (missing-self) as well as cells with expression of proteins related to cellular stress (induced-self) are selectively recognized by different NK cell receptors and are subsequently lysed [109–111]. Accordingly, NK cell effector function is tightly controlled by a dynamic balance between activating and inhibitory receptors. One of the most prominent activating receptors on NK cells is the immunoreceptor natural killer group 2, member D (NKG2D). NKG2D plays a pivotal role in NK cell maturation, effector function, and the regulation of additional activating receptors [112]. Furthermore, NKG2D is recognized to play a fundamental role in the control of experimental tumor growth, metastases and host protection from *de novo* tumorigenesis [113, 114]. Early work by Kopp and colleagues demonstrated that platelet-derived TGF-β downregulates NKG2D expression on NK cells, resulting in impaired cytotoxicity and reduced IFN-γ secretion. Notably, the exposure to platelet released cytokines also had a minor impact on other activating NK cell receptors (NKp30, NKp44, NKp46, and NKp80) [27]. Subsequent studies revealed a multilayered platelet-driven regulation of the NKG2D axis within the tumor immune microenvironment. Activated platelets alleviate the expression of NKG2D on NK cells and also the expression of the NKG2D counter-receptors MICA and MICB on ovarian and melanoma tumor cells [115]. Platelet-coated tumor cells upregulate the metalloproteinase ADAM19, promoting proteolytic shedding of the NKG2D ligands MICA and MICB from the tumor cell surface and thereby impairing NK cell-mediated cytotoxicity. Neutralization of cleaved MICA and MICB rescued NK cell function partially. Shedding of MICA and MICB not only diminishes tumor cell recognition by NK cells but also further suppresses NKG2D signaling through the accumulation of soluble ligands. Together with the TGF-β-mediated downregulation of NKG2D expression, these findings indicate that platelet-driven suppression of the NKG2D pathway is governed by redundant and reinforcing mechanisms. Adding further complexity, platelets themselves constitutively express the metalloproteinases ADAM10 and ADAM17, enabling direct cleavage of NKG2D ligands independently of platelet activation. Interestingly, ADAM10 and 17 expression was more pronounced on platelets from cancer patients than from healthy individuals, providing evidence that megakaryopoiesis can be edited or modified by tumors [116, 117]. Shedding of MICA and MICB from the tumor cells was also detectable when tumor cells were incubated with platelet releasates, demonstrating that ADAMs can be released upon platelet activation [116].

Another NK cell receptor, namely the CD94-NKG2A heterodimer, is also involved in the elimination of tumor cells. Over the past decade, extensive efforts have focused on elucidating the roles of distinct leukocyte populations within primary and metastatic tumor niches of solid malignancies [118, 119]. By contrast, direct interactions between circulating tumor cells (CTCs) and immune cells in the bloodstream remain comparatively understudied. Addressing this gap, Liu and colleagues investigated the immune interactions of disseminated pancreatic ductal adenocarcinoma cells within the hepatic portal circulation. Using complementary *in vivo* models, transcriptomic analyses, and functional assays, NK cells were identified as the principal immune effector population mediating recognition and clearance of circulating pancreatic cancer cells within the hepatic circulation. Mechanistic interrogation of the NK cell-tumor cell interactome revealed that tumor cell-expressed HLA-E confers resistance to NK cell-mediated cytotoxicity. On NK cells, engagement of the inhibitory CD94-NKG2A heterodimer by HLA-E suppresses cytotoxic effector function [120]. Interestingly, this inhibitory HLA-E-CD94-NKG2A axis operates selectively in circulating tumor cells but not in established metastatic lesions. Accordingly, the HLA-E-CD94-NKG2A dyad constitutes a circulation-specific immune checkpoint facilitating immune evasion of CTCs. To elucidate the regulation of HLA-E expression, RGS18 was identified as selectively upregulated in circulating tumor cells, but not in primary tumors or metastatic nodules. Unexpectedly, RGS18 was transferred from platelets adhering to or internalized by circulating tumor cells. Importantly, uptake was restricted to platelets derived from tumor-bearing hosts. Platelet-derived RGS18 attenuates Akt phosphorylation, resulting in stabilization of glycogen synthase kinase 3β (GSK3β) and enhanced nuclear activity of CREB1. Activated CREB1 directly binds the HLA-E promoter, thereby driving elevated HLA-E expression on tumor cells. Collectively, these findings reveal a platelet-mediated mechanism by which circulating tumor cells evade NK cell-mediated clearance through intercellular transfer of RGS18 and subsequent induction of the inhibitory immune checkpoint ligand HLA-E.

Beyond the above mentioned NKG2 receptor family, NK cell reactivity is controlled by multiple further activating and inhibitory receptor-ligand systems. An activating receptor is CD226 that is also known as DNAM-1 (DNAX accessory molecule-1) and that also acts as an adhesion receptor for NK, T cells and monocytes [121, 122]. CD226 functions as a potent activator of NK cell cytotoxicity across hematopoietic and non-hematopoietic tumor models [123]. Upon ligand engagement, CD226 is phosphorylated by the Src family kinase Fyn and cooperates with the integrin LFA-1 to enable downstream signaling and cellular activation [122, 124]. Notably, CD226 is also expressed on platelets and contributes to platelet aggregation and secretion, although the underlying signaling mechanisms remain incompletely defined [125]. In the course of tumor cell interaction, CD226 recognizes CD155 (the receptor for the poliovirus, Necl-5) and CD112 (Nectin-2) which are upregulated on different tumor entities e. g. melanoma, leukemia and colon cancer cells [122, 126, 127]. Through recognition of the stress-induced ligands CD155 and CD112, CD226 serves as a central tumor surveillance receptor on NK cells. Another NK cell receptor that binds CD155 with high affinity is CD96 that is also known as T cell-activated increased late expression (Tactile). CD96 was initially proposed to promote NK cell adhesion to target cells and enhance cytotoxicity upon ligand engagement [128]. In T cells, CD96 has a co-stimulatory role in activation and effector function. However, subsequent studies revealed that CD96 competes with CD226 for shared ligands and exerts inhibitory effects on NK cell function [129]. This seems plausible since the cytoplasmic part of CD96 contains an immunoreceptor tyrosine-based inhibition motif (ITIM) which is able to transduce inhibitory signals [130, 131]. These findings underscore the complexity of NK cell receptor signaling, which is further shaped by platelet interactions. Melanoma and ovarian cancer cells cloaked with platelets reveal a significant downregulation of NK-cell activating receptors CD112 and CD155 [115]. Concomitantly, exposure of NK cells to platelets or platelet releasates resulted in reduced surface expression of CD226 and CD96. Recombinant TGF-β also suppressed CD226 expression and TGF-β neutralization in platelet releasate partially inverted CD226 suppression. These observations implicate platelet-derived TGF-β as a key mediator of NK cell receptor downregulation. Nevertheless, additional platelet-derived factors are likely to contribute to this regulatory process. This assumption is supported by the finding that TGF-β alone significantly increased CD96 expression [115]. This differential regulation highlights receptor-specific effects of TGF-β signaling on NK cells. Collectively, these data indicate that platelets broadly remodel NK cell receptor repertoires, extending beyond the NKG2D/NKG2A-CD94 and CD226/CD96 axes.

**Fig. 4:**
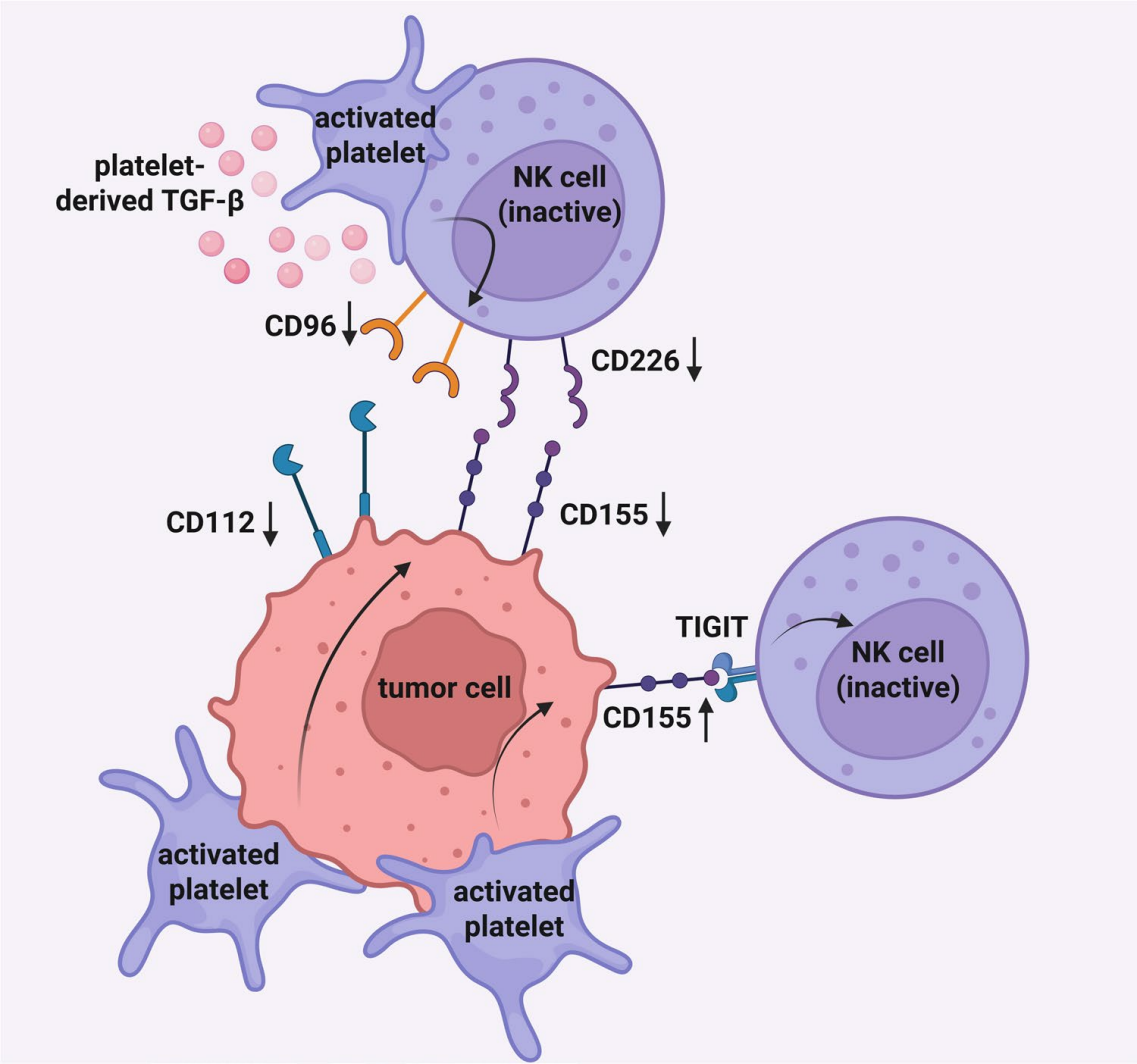
Activated platelets modulate natural killer (NK) cell function by altering the expression of activating receptor–ligand pairs. Platelets have been shown to reduce the expression of the activating receptors CD226 and CD96 on NK cells, as well as their corresponding ligands CD112 and CD155 on tumor cells, thereby impairing NK cell-mediated cytotoxicity. In addition, context-dependent upregulation of CD155 on tumor cells has been reported, which promotes engagement of the inhibitory receptor TIGIT (T cell immunoreceptor with immunoglobulin and ITIM domains) on NK cells. This interaction further suppresses NK cell activity and contributes to tumor immune evasion

In contrast to the above described platelet-induced downregulation of CD155 observed in certain tumor contexts, Sun and colleagues could recently show that a direct interaction between platelets and liver cancer cells leads to an upregulation of CD155 on the liver cancer cells by triggering the FAK/JNK/c-Jun signaling cascade [132]. The major NK cell receptor that ligates to CD155 was identified as T cell immunoreceptor with immunoglobulin and ITIM domain (TIGIT) but interestingly neither CD226 nor CD96. TIGIT is regarded as an immune checkpoint molecule and is overexpressed on different immune cell entities in the tumor microenvironment across diverse malignancies [133, 134]. It has been associated with NK cell exhaustion and a general immunosuppressive microenvironment [135, 136]. Platelet-induced enhancement of the CD155-TIGIT interaction attenuated NK cell cytotoxicity, thereby enabling circulating tumor cells to evade immune surveillance (Fig. 4). A disruption of the CD155-TIGIT interaction by TIGIT blockade restored NK-mediated CTC killing and impeded tumor metastasis [132]. Thus, platelets emerge as context-dependent regulators of immune checkpoint expression, with tumor-specific cues determining whether checkpoint pathways are enhanced or suppressed.

Glucocorticoid-induced TNF-related protein ligand (GITRL), also known as TNFSF18 or TL6, belongs to the TNF superfamily and is expressed on megakaryocytes and in platelet granules [137]. The GITRL receptor GITR is expressed on human T-, NK and myeloid cells and is involved in the regulation of the immune response [138, 139]. Functionally, the GITR-GITRL axis exerts context-dependent immune effects: while GITR ligation enhances antitumor responses in murine T cells, engagement of GITR on human NK cells suppresses cytotoxic activity [140, 141]. Accordingly, the GITR-GITRL dyad is increasingly recognized as an immune checkpoint pathway. In platelets, GITRL is translocated to the plasma membrane upon activation but does not appear to trigger reverse signaling affecting platelet degranulation. As mentioned above, activated platelets adhere to tumor cells and cloak their surface, resulting in a pseudoexpression of platelet-derived membrane proteins, including GITRL. This platelet-derived GITRL shield impairs NK cell cytotoxicity and IFNγ secretion, thereby protecting tumor cells from NK cell-mediated destruction. Whether this transfer of platelet proteins to tumor cells involves trogocytosis or alternative membrane exchange mechanisms remains to be clarified.

Clinically, breast cancer patients exhibit a higher fraction of GITRL-positive platelets compared with healthy individuals. Interestingly, GITRL-positive platelets were most abundant in intermediate-stage disease (T2) compared with early (T1) or advanced stages (T3–T4) [142]. Moreover, the proportion of GITRL-expressing platelets inversely correlated with the occurrence of metastatic disease. These findings suggest that platelet-derived GITRL may serve as a potential biomarker for disease staging and metastatic risk. To explore the mechanisms underlying increased platelet GITRL expression, megakaryocytes were differentiated in vitro and exposed to conditioned media from breast cancer cell lines. Elevated platelet GITRL expression was observed particularly in response to tumor-derived factors enriched in TGF-β [142]. These studies identify platelet-derived GITRL as a checkpoint-like regulator of NK cell activity that may contribute to tumor immune escape while simultaneously serving as a potential prognostic biomarker in cancer.

Collectively, these findings underscore the remarkable complexity by which platelets regulate NK cell-mediated antitumor immunity. Whether therapeutic targeting of this intricate platelet-NK cell-tumor cell axis can be translated into clinically effective strategies remains an open question and will require rigorous validation in preclinical models and, ultimately, in patients.

### TLT-1 as a platelet-specific immune modulator in cancer

Triggering receptor expressed on myeloid cell-like (TREM-like) transcript-1 (TLT-1) is uniquely expressed in platelets and megakaryocytes [143]. Structurally, TLT-1 comprises an extracellular IgV-like domain, a transmembrane region, and two cytoplasmic ITIM motifs [144]. TLT-1 is located in α-granules of resting platelets and shifted to the platelet surface upon activation [145]. Platelet activation also triggers the release of soluble fragments of TLT-1 into the serum [146]. Accordingly, elevated circulating TLT-1 levels may reflect systemic platelet activation in pathological conditions. Functional studies demonstrated that antibody-mediated blockade of TLT-1 impairs thrombin-induced platelet aggregation, underscoring its role in hemostasis and thrombus formation [147]. Mechanistically, TLT-1 associates with ezrin/radixin/moesin (ERM) cytoskeletal adaptor proteins, providing a structural basis for its pro-aggregatory function despite the presence of ITIM motifs [148]. Extracellularly, TLT-1 binds to fibrinogen thereby supporting clot formation by linking fibrinogen to the platelet cytoskeleton via ERM family proteins. Consistently, TLT-1-deficient mice exhibit prolonged bleeding times and aggravated inflammatory hemorrhage following lipopolysaccharide challenge. These findings suggest a protective role for TLT-1 in maintaining vascular integrity during systemic inflammation [148]. Beyond hemostasis, TLT-1 regulates platelet-neutrophil aggregate formation, promotes neutrophil recruitment to inflamed tissues, and enhances neutrophil survival [149, 150]. Until recently, its relevance in tumor-associated immune regulation remained unclear. Tyagi et al. identified a distinct platelet phenotype in non-small cell lung cancer (NSCLC) patients which was associated with increased mitochondrial integrity, reduced apoptosis and enhanced expression of P-selectin and TLT-1 [151]. Further, TLT-1 surface expression raised linearly with tumor size in mice. These observations suggest that TLT-1 expression correlates with tumor burden and platelet reprogramming in cancer. Given its soluble form and IgV-domain structure, TLT-1 was hypothesized to directly interact with immune cells [151]. Injection of recombinant TLT-1 into mice revealed a binding of TLT-1 to T cells. Dose-dependent administration of recombinant TLT-1 reduced the proportion of peripheral IFNγ⁺ CD8⁺ T cells. Importantly, this effect was selective for IFNγ^+^ CD8⁺ T cells, while CD4⁺ T cells remained largely unaffected. *In vivo*, TLT-1 administration promoted tumor growth and diminished tumor-infiltrating CD8⁺ T cells, indicating suppression of cytotoxic antitumor immunity. Conversely, antibody-mediated TLT-1 blockade restrained tumor growth and restored CD8⁺ T cell effector responses. In NSCLC patients, higher TLT-1 levels correlated with reduced survival and T cell exhaustion in the tumor tissue. Mechanistically, TLT-1 was shown to bind the CD3ε subunit of the T cell receptor complex, activating NF-κB signaling and promoting an activation-induced cell death-like phenotype in CD8⁺ T cells. Ex vivo platelet-T cell co-culture experiments using patient-derived cells confirmed that TLT-1 induces CD8⁺ T cell apoptosis, whereas its blockade reinstates antitumor immunity. Collectively, these findings identify TLT-1 as a platelet-derived immune checkpoint-like molecule that selectively impairs CD8⁺ T cell-mediated tumor control at least in mice. Whether TLT-1 upregulation represents a generalized feature of tumor-educated platelets across malignancies and whether therapeutic targeting of TLT-1 in patients is feasible in cancer or inflammatory disorders warrants further investigation.

## Concluding remarks

This review summarizes emerging evidence that positions platelets as active regulators of tumor-immune cell communication in cancer. The different mechanisms are summarized in Table 1. Collectively, these findings reveal a complex network of platelet-mediated mechanisms that potentially modulate multiple immune effector cell populations within the tumor microenvironment. However, a substantial proportion of these findings originates from reductionist in vitro models that do not fully recapitulate the cellular and molecular complexity of the *in vivo* tumor microenvironment. Consequently, the physiological relevance and relative contribution of these mechanisms under *in vivo* conditions may differ and require further validation in more complex experimental systems.

**Table 1:** Overview of the platelet-mediated immune modulation mechanisms

Platelet-derived/-affected receptors/molecules	Affected Immune Cell entity	Mechanism of immune escape	Reference
PD-L1	Cytotoxic T cells	• Platelet EGF & serotonin upregulate PD-L1 on TCs = > immune evasion • Transfer of PD-L1 from TCs to platelets • Transfer of PD-L1 from platelets to TCs • PD-1 & CTLA-4-presenting platelets	[37–41]
Erbin	Plasma & cytotoxic T cells	• Platelet Erbin deficiency = > increases mitochondrial activity = > platelet palmitoyl-L-carnitine secretion = > CD138⁺ plasma cell differentiation = > increased frequency of IFNγ⁺ and perforin⁺ T cells = > reduced CRC metastasis in mice	[51]
TXA_2_	Cytotoxic T cells/tumor microenvironment	• Platelet TXA_2_ inhibits robust T-cell activation & cytokine production/TXA_2_ induces platelet-tumor cell clot formation & recruitment of pro-metastatic myeloid cells	[64, 65]
CD40L	Cytotoxic T cells	• Platelet CD40L triggers anti-liver cancer immune response	[75]
TGF-β	Cytotoxic T cells, NK cells, neutrophils, MDSCs	• Platelet TGF-β reduces CD8⁺ and NK cell cytotoxicity & converts conventional CD4^+^ to Treg cells • Platelet TGF-β reduces neutrophil recruitment • Platelet TGF-β induces expansion of MDSCs	[89–91]
CD39, CD73	Cytotoxic T cells	• Platelets upregulate CD39 & CD73 on TCs leading to ATP-adenosine conversion and reduced cytotoxic T cell activity & expression of inhibitory receptors (i. e. PD-1)	[106–108]
NKG2D	NK cells	• Shedding of NKG2D ligands MICA and MICB by platelet-induced ADAM19 & platelet-expressed ADAM10 & ADAM17	[116, 117]
CD94-NKG2A	NK cells	• Platelet-derived RGS18 upregulates HLA-E in CTC leading NK cell evasion	[120]
CD226/CD96/TIGIT	NK cells	• Platelet-mediated downregulation of activating CD112 & CD155 receptors on melanoma & ovarian cancer cells • Platelet-mediated downregulation of CD226 & CD96 on NK cells • Upregulation of CD155 on liver cancer cells, which binds to TIGIT on NK cells & reduces NK cell activity	[115, 132]
GITR ligand	NK cells	• Platelet expressed GITR ligand binds to GITR & mitigates NK cell cytotoxicity & IFNγ secretion	[137, 142]
TLT-1	IFNγ^+^ CD8⁺ T cells	• Platelet-derived TLT-1 binds to the CD3ε subunit on CD8⁺ T cells and leads to T cell apoptosis	[151]

The majority of the described pathways converge on the suppression of antitumor immunity and thereby promote tumor progression and metastatic dissemination. Mechanistically, platelets exert these immunomodulatory effects through direct receptor-ligand interactions, secretion of soluble mediators, and enzymatic generation of immunosuppressive metabolites such as adenosine. These observations raise the possibility that pharmacological platelet inhibition may enhance the efficacy of conventional chemotherapy or immune checkpoint blockade. However, pharmacological inhibition of platelet function inevitably carries the risk of bleeding complications, which may substantially limit its therapeutic applicability and compromise patient safety. Importantly, the mechanisms described to date likely represent only a fraction of the platelet-mediated regulatory circuits shaping tumor immune surveillance. Notably, additional studies have described immunomodulatory effects of platelet-derived receptors and mediators in diverse immune contexts; however, their specific contribution to antitumor immunity remains to be established [152]. For example, platelet-derived PF4 has been implicated in the generation of pro-tumorigenic TH_1_-like regulatory T cells [153], while platelet-derived TGF-β may enhance the suppressive activity of MDSCs [154]. Platelets also actively modulate innate immune responses to facilitate tumor cell survival and metastatic progression. Labelle and colleagues demonstrated that tumor cell-activated platelets secrete the chemokines CXCL5 and CXCL7, which mediate rapid granulocyte recruitment to circulating tumor cells and thereby promote the establishment of early metastatic niches. Pharmacological blockade of the corresponding receptor CXCR2 or transient depletion of platelets or granulocytes impaired early niche formation and significantly reduced metastatic seeding [20]. Similarly, Läubli and colleagues showed that cooperative interactions between platelets, leukocytes, and tumor cells activate microvascular endothelial cells to release the chemokine CCL5. Subsequent CCL5-mediated monocyte recruitment further supports the generation of a permissive metastatic microenvironment [155]. In addition, platelets can promote M2-like macrophage polarization, thereby contributing to an immunosuppressive tumor microenvironment in bladder cancer [156]. Together, these findings highlight that platelets not only protect tumor cells from adaptive immune elimination but also actively orchestrate the recruitment of innate immune cells to establish metastatic-supportive niches. Consequently, substantial gaps remain in understanding whether these platelet-mediated immune regulatory pathways are conserved across tumor entities and disease stages.

Simultaneous targeting of multiple platelet-derived immunosuppressive pathways may therefore represent a potential strategy to reinforce antitumor immunity. However, such interventions may also carry substantial risks, particularly bleeding complications, which could limit their clinical applicability. Accordingly, several variables will need to be carefully considered, including the timing of platelet inhibition relative to concomitant therapies (such as immune checkpoint inhibitors, kinase inhibitors, or chemotherapy), tumor-specific biology, and individual patient characteristics, in order to define safe and effective therapeutic windows. This consideration is particularly important given emerging evidence that platelet inhibition may also impair CD8⁺ T cell-mediated immunity, for example through reduced platelet-derived CD40L signaling. Thus, the development of therapeutic approaches that selectively target the immunomodulatory functions of platelets—while sparing their essential role in hemostasis and CD40L activity—may enable more precise and safer modulation of platelet-driven immune regulation in cancer than a platelet inhibition with currently established drugs.

## Data Availability

No datasets were generated or analysed during the current study.
